# Quad Fever After a Traumatic Cervical Spinal Cord Injury: Diagnostic Challenges and Therapeutic Response to Bromocriptine

**DOI:** 10.7759/cureus.101000

**Published:** 2026-01-07

**Authors:** Rita Bragança, Mariana Esteves, Paulo Carrola

**Affiliations:** 1 Internal Medicine, Unidade Local de Saúde de Trás-os-Montes e Alto Douro, Vila Real, PRT

**Keywords:** hyperpyrexia, neurogenic fever, quadriplegia, spinal cord injuries, thermodysregulation

## Abstract

Quad fever is a rare, potentially fatal form of neurogenic hyperpyrexia occurring after high cervical or upper thoracic spinal cord injury (SCI) and is frequently misdiagnosed as severe infection. It is characterised by extreme, non-infectious hyperthermia resulting from autonomic and central thermoregulatory dysfunction. We report the case of a previously healthy 72-year-old woman who sustained a traumatic C4-C5 SCI and developed abrupt postoperative hyperpyrexia (40.9 °C) within 12 hours of anterior cervical decompression and fusion. Despite antipyretics, broad-spectrum antibiotics, and physical cooling, daily high-grade fevers persisted. An extensive evaluation excluded infectious, inflammatory, endocrine, neoplastic, and hardware-related causes, with normal inflammatory markers and sterile cultures. Based on the clinical context and exclusion of alternative etiologies, a diagnosis of quad fever was established. Enteral bromocriptine 5 mg twice daily was initiated based on its dopaminergic modulation of hypothalamic thermoregulation, resulting in complete defervescence within 24 hours and sustained apyrexia thereafter, without adverse effects. This allowed the patient to continue a structured rehabilitation programme and be discharged in good general condition. This case underlines quad fever as a diagnosis of exclusion in patients with severe SCI and refractory fever, illustrates the potential morbidity and resource use associated with delayed recognition, and supports the role of bromocriptine as a promising therapeutic option.

## Introduction

Acute spinal cord injury (SCI) remains a life-threatening event, radically transforming mobility and sensation. Among the less frequently recognised complications of high cervical or upper thoracic SCI is a phenomenon known colloquially as quad fever - defined as an extreme, non-infectious hyperpyrexia (often exceeding 40.8 °C) occurring in individuals with quadriplegia or high paraplegia following SCI [1,2].

Despite heterogeneous definitions, uncertain etiology, and the limited number of reported cases, quad fever is consistently associated with a substantially increased mortality, with reported rates reaching up to 28.6% [1,3].

Unlike typical febrile responses driven by infection or inflammation, this central thermoregulatory failure appears to stem from autonomic dysfunction and impaired hypothalamic-spinal pathways, rendering traditional antipyretic strategies largely ineffective [1,4].

Although fever of any cause is frequent after SCI - reported in up to 60% of patients - the neurogenic or non-infectious subset remains under-recognised, with estimated incidences ranging from 2.6% to 27.8% [4,5].

What complicates management is that quad fever is often a diagnosis of exclusion [4]. Patients typically present with persistent high-grade fever, negative microbiological studies, and no identifiable infectious cause. Consequently, misdiagnosis is common, leading to unnecessary antibiotic use and delayed recognition of the true thermoregulatory dysfunction [6].

In light of these challenges, this article aims to present a detailed clinical case of quad fever following high cervical traumatic SCI, outlining diagnostic reasoning, therapeutic interventions, and outcomes.

## Case presentation

A 72-year-old woman with no relevant past medical history was admitted to the emergency department after an accidental fall from approximately 2 m, resulting in head and neck trauma. On physical examination, her Glasgow Coma Scale (GCS) score was 15, and vital signs were within normal limits, with the patient afebrile. Cardiopulmonary auscultation was unremarkable. The initial neurological assessment revealed acute flaccid tetraparesis (Medical Research Council Muscle Scale 0/5) below the C4 motor level and C5 sensory level; deep tendon reflexes were absent, and plantar reflexes were bilaterally absent.

**Figure 1:**
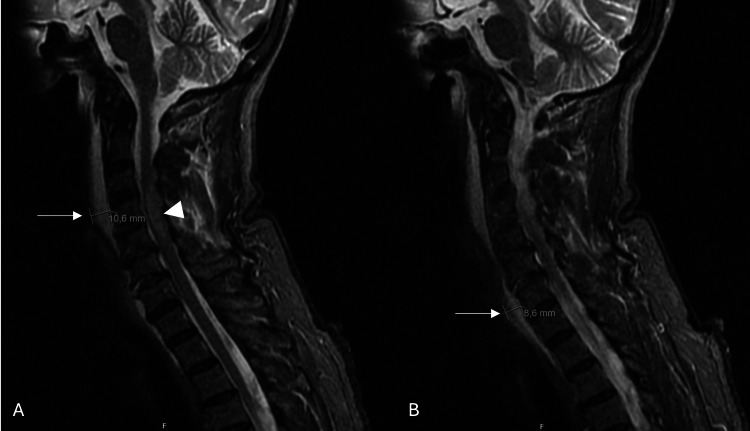
Sagittal T2-weighted MR images of the cervical spine. (A) and (B) demonstrate a prevertebral hematoma anterior to the mid-cervical vertebral bodies, with maximal thickness measurements of 10.6 mm and 8.6 mm in different sagittal planes (arrows), and associated spinal cord edema/contusion (arrow tip) at the C4–C5 level.

She underwent magnetic resonance imaging (MRI) of the head and neck, which revealed prevertebral hematomas at C2 and C5, likely related to injury of the anterior longitudinal ligament, as well as a linear fracture of the anteroinferior aspect of the C4 vertebral body with minimal adjacent bone edema. There was also evidence of a C6-C7 disc rupture and mild expansion of the spinal cord at C4-C5, consistent with spinal cord edema/contusion (Figure 1). A diagnosis of American Spinal Injury Association (ASIA) Impairment Scale grade A spinal cord injury was made, and she was transferred to a tertiary neurosurgical unit, where she underwent anterior cervical decompression and fusion at C4-C5 using a titanium plate and an iliac crest autograft. The procedure was uneventful.

**Figure 2:**
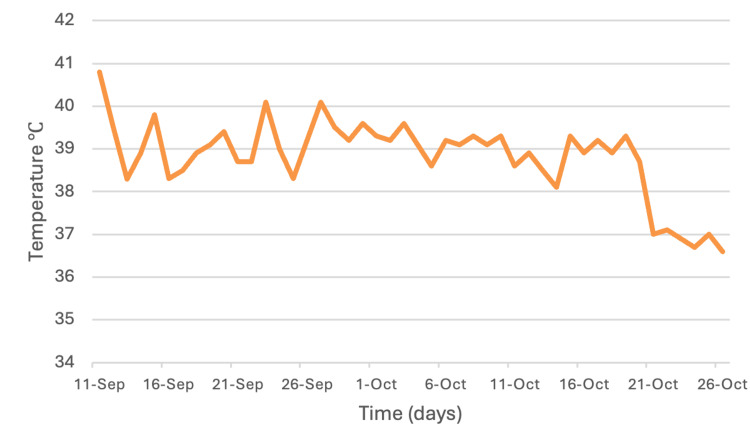
Daily maximum temperature trend during hospitalization. Line graph showing the daily maximum recorded temperatures between September 11 and October 26, 2021. The patient initially presented with marked hyperpyrexia, with temperatures exceeding 40 °C, followed by a persistent pattern of high fever consistent with non-infectious fever after cervical spinal cord injury. Bromocriptine 5 mg twice daily was initiated on October 20, after which a progressive decline in temperature was observed, with resolution of fever in the days that followed.

Twelve hours postoperatively, she developed a fever of 40.9 °C, for which empirical broad-spectrum antibiotic therapy with piperacillin-tazobactam and vancomycin was initiated; however, the fever persisted. Antipyretic agents were ineffective, and the only interventions that produced partial defervescence were physical cooling measures, including ice packs and cold bladder irrigation. Due to ongoing daily fever spikes (Figure 2), an internal medicine consultation was requested.

During hospitalization, there was no evidence of cough, chest pain, dyspnoea, gastrointestinal or genitourinary symptoms, nor any history of recurrent fever, arthralgia, rash, xerostomia, xerophthalmia, oral ulcers, asthenia, anorexia, weight loss, or night sweats. She reported no relevant epidemiological exposures or recent drug use. On examination, she was haemodynamically stable, with no episodes of dysautonomia reported and no altered mental status (GCS 15). The remainder of the physical examination was unremarkable, including no lymphadenopathy, cutaneous rashes, mucosal enanthems, or clinical signs of arthritis.

Given the postoperative fever of undetermined origin, a stepwise diagnostic workup for infectious, inflammatory, and neoplastic causes was undertaken. Drug-related hyperthermia was ruled out.

Hematologic and inflammatory studies revealed a normocytic anemia on blood smear, with C-reactive protein, erythrocyte sedimentation rate, and procalcitonin remaining within normal limits. Peripheral blood immunophenotyping demonstrated a small lymphoid population without immunophenotypic abnormalities, providing no evidence of an underlying lymphoproliferative disorder.

Biochemical evaluation showed normal ACE, complement fractions (C3c and C4), creatine kinase, serum iron, transferrin saturation, triglycerides, vitamin B12, and β2-microglobulin. Immunoglobulin A, G, and M levels were also within the normal range, whereas immunoglobulin E was increased. Serum protein electrophoresis did not reveal any monoclonal component. Iron studies showed slightly elevated serum ferritin with reduced total iron-binding capacity (Table 1). Comprehensive microbiological evaluation of blood and urine, including fungal and mycobacterial studies, was also negative.

**Table 1:** Hematology, biochemistry, and endocrine results. Abbreviations: C3c, complement component 3c; C4, complement component 4; ESR, erythrocyte sedimentation rate; Ig, immunoglobulin; IgA, immunoglobulin A; IgE, immunoglobulin E; IgG, immunoglobulin G; IgM, immunoglobulin M; ACTH, adrenocorticotropic hormone; TSH, thyroid-stimulating hormone; UI, international units

Parameter	Result	Reference value
Hematology/Inflammation		
Blood smear	Normocytic anemia	—
C-reactive protein	<0.5 mg/dL	<0.5 mg/dL
ESR	20 mm/1st h	0–30 mm/1st h
Hemoglobin	10.9 g/dL	12–16 g/dL
Peripheral blood immunophenotyping	Small-sized lymphoid population without immunophenotypic alterations suggestive of lymphoproliferative disease	—
Procalcitonin	0.1 ng/mL	<0.5 ng/mL
White blood cells	6.59×10³/µL	4.00–11.00×10³/µL
Neutrophils	4.44×10³/µL	2.00–7.50×10³/µL
Lymphocytes	1.60×10³/µL	1.50–4.00×10³/µL
Monocytes	0.40×10³/µL	0.20–0.80×10³/µL
Eosinophils	0.11×10³/µL	0.04–0.40×10³/µL
Basophils	0.05×10³/µL	0.02–0.10×10³/µL
Biochemistry/Metabolism		
ACTH	58.2	< 63.3 ng/L
Angiotensin-converting enzyme	29 U/L	6–52 U/L
C3c	126 mg/dL	90–180 mg/dL
C4	25 mg/dL	12–36 mg/dL
Creatine kinase	104	—
Free thyroxine (free T4)	17.2 pmol/L	8.2–21 pmol/L
Free triiodothyronine (free T3)	3.2 pmol/L	3.1–6.8 pmol/L
IgA	910 mg/dL	600–1570 mg/dL
IgE	847 (↑) UI/mL	<100 UI/mL
IgG	271 mg/dL	50–373 mg/dL
IgM	101 mg/dL	40–325 mg/dL
Serum ferritin	312 ng/mL	15–150 ng/mL
Serum iron	64 µg/mL	37–145 µg/mL
Serum protein electrophoresis	No monoclonal peaks	—
Total iron-binding capacity	164 mg/dL	228–360 mg/dL
Transferrin saturation	39%	>20%
Triglycerides	129 mg/dL	<150 mg/dL
TSH	1.25 mIU/L	0.27–4.2 mIU/L
Vitamin B12	219 pg/mL	191–663 pg/mL
β2-microglobulin	1.3 µg/mL	0.8–2.2 µg/mL

Extended serologic testing indicated past exposure to hepatitis A virus, cytomegalovirus, and Epstein-Barr virus. Human immunodeficiency virus (HIV) type 1 and type 2 (HIV-1 and HIV-2) antibodies were non-reactive. Tests for rickettsial diseases, typhoid fever, brucellosis (Widal, Wright, Rose Bengal), and toxoplasmosis immunoglobulin M (IgM) were negative. A peripheral blood zoonosis PCR panel (*Anaplasma*, *Ehrlichia*, *Borrelia*, *Babesia*, *Coxiella*, *Rickettsia*) was likewise negative. The interferon-gamma release assay for tuberculosis was negative. Autoimmune screening yielded negative results (Table 2). 

**Table 2:** Immunology and autoimmunity results. Abbreviations: dsDNA, double-stranded DNA; ENA, extractable nuclear antigens; FEIA, fluorescent enzyme immunoassay; Ig, immunoglobulin; IgG, immunoglobulin G; IgM, immunoglobulin M; IIF, indirect immunofluorescence; Mi-2, myositis-specific autoantigen Mi-2; PCNA, proliferating cell nuclear antigen; PM-Scl, polymyositis–scleroderma overlap antigen; RF, rheumatoid factor; RNA Pol III, RNA polymerase III; RNP, ribonucleoprotein; Scl-70, DNA topoisomerase I (Scl-70 antigen); SS-A/Ro, Sjögren’s-syndrome-related antigen A; SS-B/La, Sjögren’s-syndrome-related antigen B; UI, international units

Parameter	Result	Reference value
Immunology/Autoimmunity		
Anti-cardiolipin antibodies (IgG/IgM)	Negative	—
Anti-myeloperoxidase antibody	<0.2 U/mL	<3.5 U/mL
Anti-phospholipid antibodies (IgG/IgM)	Negative	—
Anti-thyroglobulin	<12	<12
Anti–dsDNA antibody (IIF)	<1:10	<1:10
Anti–proteinase 3 antibody	<0.2 U/mL	<3 U/mL
Antineutrophil cytoplasmic antibodies	<1:20	<1:20
Antinuclear antibodies (IIF)	<1:80	<1:80
Extractable nuclear antigen (ENA) screen (dsDNA, SS-A/Ro, SS-B/La, Sm, RNP, Scl-70, centromere, Jo-1, fibrillarin, RNA Pol III, PM-Scl, PCNA, Mi-2, ribosomal P) (FEIA)	Negative	Negative
Rheumatoid factor	4 UI/mL	<14 UI/mL

Chest radiography and contrast-enhanced computed tomography (CT) of the chest were unremarkable, with no evidence of pulmonary embolism. Abdominal and pelvic CT revealed no abscesses or intra-abdominal foci. Transthoracic echocardiography showed normal valvular structures without vegetations. Duplex ultrasonography of the lower extremities excluded deep vein thrombosis. A lumbar puncture was performed, with no evidence of central nervous system infection.

Bone marrow aspirate and biopsy showed slightly hypocellular smears diluted with peripheral blood but no morphological abnormalities; Leishmania testing was negative. A comprehensive zoonosis panel (*Anaplasma*, *Ehrlichia*, *Borrelia*, *Babesia*, *Coxiella*, *Rickettsia*, tick-borne encephalitis virus) was negative, and bone marrow cultures were sterile. Flow cytometry revealed a small lymphoid population without immunophenotypic abnormalities suggestive of a lymphoproliferative disorder. The bone core was scant but demonstrated granulocytic hypoplasia without additional morphologic alterations.

Because of the elevated serum IgE, hypersensitivity to the titanium implant was considered. Standardised epicutaneous patch testing with a metals panel, including titanium, yielded negative results.

**Figure 3:**
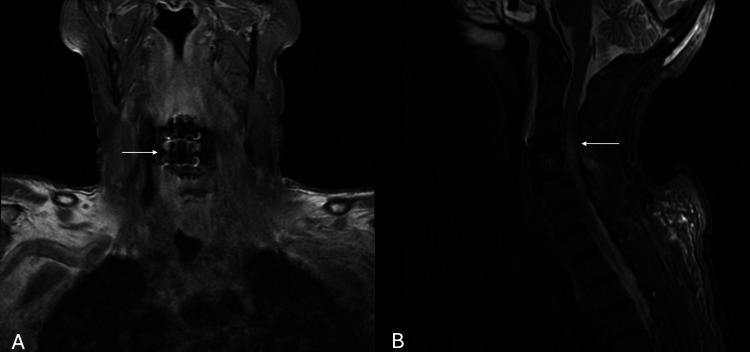
Postoperative magnetic resonance imaging of the cervical spine. (A) Coronal T1-weighted image demonstrating expected postoperative changes following anterior cervical decompression and fusion, without evidence of epidural collection, spondylodiscitis, or hardware-related complications (arrow). (B) Sagittal STIR-weighted image showing hyperintense signal within the spinal cord at the C4–C5 level, consistent with spinal cord edema/contusion (arrow).

Repeat cervical MRI performed postoperatively demonstrated expected postoperative changes, without evidence of spondylodiscitis, epidural collection, or hardware complications (Figure 3). Fluorodeoxyglucose positron emission tomography (FDG-PET) demonstrated isolated homogeneous splenic hypermetabolism without splenomegaly, interpreted as reactive reticuloendothelial activity rather than occult malignancy.

A diagnostic naproxen challenge did not produce defervescence, making neoplastic fever less likely. A five-day course of oral corticosteroids also failed to modify the temperature curve. Given the early postoperative onset, persistent fever despite antipyretics, and exclusion of infectious, inflammatory, and neoplastic causes, central fever secondary to cervical spinal cord injury was diagnosed. Bromocriptine 5 mg twice daily was initiated enterally, leading to defervescence within 24 hours and sustained apyrexia thereafter. No adverse effects were observed.

The patient was discharged in good general condition. She subsequently continued a structured physiatric rehabilitation programme appropriate for traumatic spinal cord injury. At follow-up, environmental temperature control, adequate hydration, and prevention of secondary complications were emphasised.

## Discussion

Quad fever poses a diagnostic and therapeutic challenge in patients with high SCI. The present case illustrates how this rare form of neurogenic hyperpyrexia can emerge early after cervical trauma, mimic postoperative infection, and persist despite exhaustive investigation, antipyretic administration, empirical antimicrobial therapy, and physical cooling measures. In line with previous reports in which higher temperature elevations correlated with greater impairment on the ASIA scale, our case reflects this relationship precisely [1].

Recognition of this entity requires a high index of suspicion, particularly when fever remains refractory to standard interventions and no infectious, inflammatory, or neoplastic source is identified.

In our patient, the onset of hyperpyrexia within 12 hours post-surgery, coupled with the absence of local or systemic infection, normal inflammatory markers, and sterile microbiological cultures, pointed toward a central thermoregulatory origin. The extensive diagnostic workup - including serologic testing, bone marrow evaluation, advanced imaging, and exclusion of hypersensitivity reactions to titanium hardware - reflects the difficulty of confirming this diagnosis by exclusion alone. Such an approach is consistent with prior reports emphasising the need for systematic elimination of alternative etiologies before diagnosing quad fever [4,7,8].

Although the pathogenesis of quad fever has yet to be established, several hypotheses exist. Autonomic imbalance is a recognised consequence of cervical and high thoracic SCI, and disordered temperature control appears to fall within this spectrum, although the precise intermediary pathways are unclear [4]. The anterior hypothalamus serves as the central “thermostat” of the body, integrating input from thermal sensors and chemical mediators, such as cytokines and interleukins, and adjusting body temperature through vasomotor responses. When the central nervous system is damaged, disruption of the afferent and efferent connections between this region and the rest of the neuraxis may significantly impair this regulatory loop. One proposed explanation for the resulting episodes of severe hyperthermia is sympathetic dysregulation, leading to impaired vasodilation and an inability to sweat adequately at appropriate thermal setpoints [4,9].

Pharmacologic management of neurogenic hyperpyrexia remains largely empirical. Bromocriptine, a dopamine receptor agonist, was chosen in our case based on its potential to modulate hypothalamic dopaminergic activity and restore thermal balance. The rapid and sustained defervescence observed following initiation of bromocriptine supports previous observations describing its efficacy in this setting [9]. In contrast, antipyretics and corticosteroids failed to influence the temperature curve, consistent with reports indicating that central hyperthermia is unresponsive to conventional antipyretic mechanisms [2,10].

Environmental control, early recognition, and prompt initiation of targeted therapy appear to be key determinants of favourable outcomes [11]. Pharmacologic modulation, when effective, may reduce reliance on invasive strategies and improve patient comfort. Importantly, the benign postoperative appearance of the surgical site and the absence of systemic signs of infection should prompt clinicians to consider this diagnosis before escalating antibiotics unnecessarily.

The significance of early diagnosis extends beyond immediate fever control. Persistent hyperpyrexia in patients with SCI is associated with increased metabolic demand, cardiovascular instability, and secondary neuronal injury [6]. By avoiding these consequences, early recognition and appropriate treatment may improve neurological and functional recovery trajectories. Our patient’s initial stabilisation and clinical improvement after targeted therapy align with prior case reports describing favourable outcomes when quad fever is promptly identified and managed.

Nevertheless, several uncertainties remain. There are no standardised diagnostic criteria, and the true incidence of quad fever likely remains underestimated due to overlap with infectious fever syndromes. Prospective studies are needed to delineate risk factors, clarify underlying mechanisms, and establish evidence-based therapeutic protocols. Until then, clinician awareness remains the cornerstone of effective management.

## Conclusions

Quad fever remains a challenging and unsettling diagnosis, both for clinicians and for patients already facing the life-changing impact of high cervical spinal cord injury. Our case illustrates how a sustained, unexplained fever can trigger an exhausting cascade of investigations, invasive procedures, and empirical treatments before a central thermoregulatory cause is considered. Recognising this entity early - particularly when inflammatory markers are normal, cultures remain sterile, and standard antipyretics are ineffective - can help avoid unnecessary antibiotic exposure, reduce iatrogenic harm, and refocus care on what the patient most needs: comfort, stability, and a clear explanation of what is happening to their body.

At the same time, this experience highlights a broader gap in our knowledge. There are no validated diagnostic criteria, and guidance on treatment is limited to small series and case reports. In our patient, bromocriptine provided a simple, well-tolerated, and remarkably effective therapeutic option, allowing her temperature to normalise and enabling meaningful participation in rehabilitation. Larger studies are needed to clarify the mechanisms, refine diagnostic pathways, and define evidence-based management strategies. Until then, maintaining a high index of suspicion, listening carefully to the patient’s narrative, and working in close multidisciplinary collaboration remain essential to improving outcomes for individuals living through this rare but serious complication of spinal cord injury.
